# Design and Comparison of Image Hashing Methods: A Case Study on Cork Stopper Unique Identification

**DOI:** 10.3390/jimaging7030048

**Published:** 2021-03-08

**Authors:** Ricardo Fitas, Bernardo Rocha, Valter Costa, Armando Sousa

**Affiliations:** 1 Department of Mechanical Engineering, FEUP—Faculty of Engineering, University of Porto, 4200-465 Porto, Portugal; rfitas99@gmail.com (R.F.); santosrocha.bernardo@gmail.com (B.R.); 2 INEGI—Institute of Science and Innovation in Mechanical and Industrial Engineering, 4200-465 Porto, Portugal; 3 Department of Electrical and Computer Engineering, FEUP—Faculty of Engineering, University of Porto, 4200-465 Porto, Portugal; 4 INESC TEC—INESC Technology and Science (formerly INESC Porto), 4200-465 Porto, Portugal

**Keywords:** anti-counterfeiting, cork stoppers, hashing, perceptual hash, Discrete Cosine Transform, image processing, Radon transform, difference hash

## Abstract

Cork stoppers were shown to have unique characteristics that allow their use for authentication purposes in an anti-counterfeiting effort. This authentication process relies on the comparison between a user’s cork image and all registered cork images in the database of genuine items. With the growth of the database, this one-to-many comparison method becomes lengthier and therefore usefulness decreases. To tackle this problem, the present work designs and compares hashing-assisted image matching methods that can be used in cork stopper authentication. The analyzed approaches are the discrete cosine transform, wavelet transform, Radon transform, and other methods such as difference hash and average hash. The most successful approach uses a 1024-bit hash length and difference hash method providing a 98% accuracy rate. By transforming the image matching into a hash matching problem, the approach presented becomes almost 40 times faster when compared to the literature.

## 1. Introduction

The concept of Fingerprint of Things stems from an increasing trend towards the storage and analysis of a large amount of data [1]. This data is usually related to the manufacturing process and use of a given the object, creating a detailed history to which it can be attributed. This information can be very useful regarding possible improvements in production processes or quality control [1,2,3,4,5].

It is often referred to as a possible solution in the fight against the counterfeiting of goods [3]. Regarding this point, one of the most pressing issues concerns the counterfeiting of medicines and other medical products. According to the World Health Organization (WHO) [6,7], this is a considerable problem and one which has increased in both dimension and implication, with a negative impact at the economic and social level, as well as raising health concerns, both for individuals and the public. As an example, the WHO mentions, on this point and by way of example, the contribution of this type of fraud to an increase antimicrobial resistance.

The unique identification of these products and the storage of a maximum of information associated with them, have emerged as a possible solution to this problem. This leads to a direct link between the manufacturer and the consumer that ideally would not be interrupted by entities with malicious motives and would thus serve as a guarantee of the product’s reliability and quality. For this purpose, some methods have already been put into practice such as bar codes, Quick Response (QR) codes, Radio-Frequency IDentification (RFID) tags, Near Field Communication (NFC) technology, and even serial numbers, among others. However, they do not constitute fully functional options, not only because they can be easily manipulated and cloned but also because, in their vast majority, they identify the packaging instead of the product itself [8,9,10].

Although these are steps towards solving the problems presented, they raise a series of issues, regarding safety and cost [1]:

- They can undergo several tampering attempts such as interception of communication, data corruption, forgery, and cloning.
- Systems as simple as serial numbers or bar codes are quite simple to forge or reproduce.
- Small components produced in large quantities, such as screws, would be made more expensive by the insertion, in each of them, of an RFID or NFC tag or other identification object. For even smaller components, it may be impossible to incorporate any type of identifier.
- The combination of label materials with those of the component could lead to obstacles at recycling.

As a way to respond to these challenges, the new Fingerprint of Things systems has emerged. It does not require direct contact with the object to identify it, thus constituting a non-invasive procedure. Moreover, the fact that it does not require any type of tag or sensor, relying exclusively on specific characteristics of the object as a way of detection, provides a solution to most of the issues which were raised earlier. In addition, since it includes hardly any type of additional processing or information attached to the product, it can be quickly coupled to a production line, requiring only a station that collects the elements relevant of the product and stores them in a database.

A question now arises. What kind of objects can this method identify? In this regard, it is important to refer to the concept of Physical Unclonability and Disorder (PUD). PUD [11] security systems use randomness in the distribution of certain physical characteristics of an object to identify it, and can be divided into two categories: UNique Objects [10] (UNOs) and Physical Unclonable Functions (PUFs) [10,12,13].

UNOs are physical objects with unique and measurable characteristics that must be difficult, if not impossible, to clone, given the great randomness of the system. Furthermore, these systems must be robust as to preserve those characteristics in the face of normal conditions of use and ageing for relatively long periods of time. PUFs are constituted by physical systems that are subject to certain stimuli. In view of these stimuli, the PUF must generate a unique response. PUF must be unpredictable and unidirectional [14].

It has been proved that cork stoppers are unique objects [10] with characteristics that allow their classification as UNOs. Costa et al. [15] suggested a method based on clustering and matching images with the Recognition of Individual Objects using Tagless Approaches (RIOTA) algorithm. The proposed implementation and its use of key point descriptors lead to a considerable computational cost involved in matching descriptors from the two images to compare, resulting in impractical search times. This issue is to be addressed in the present work, in which possible methodological solutions are suggested and tested. Some concern was also expressed regarding the influence of image quality in detecting correct matches. Image quality is affected by noise, rotation, cropping, scaling, and illumination. Among these, rotation is presumed to be one of the main problems and is tackled as well.

The proposed work aims to find an alternative solution for the authentication of cork stopper representations, using hash codes instead of images. Therefore, it does as follows:

- allows for the reduction in noise and non-relevant data at an early stage of the process;
- tests and compares a set of hashing methods and image-processing techniques for the treatment of cork stopper images;
- proposes the optimized design for the cork stopper authentication process.

This paper is structured as follows: the state-of-the-art, image hashing method developments in the literature are reviewed and classified; in the background of concepts section, some known methods about image processing and data compressing is discussed; the tests performed on the methods for obtaining the correspondent success rate are described in the methodology section; in the results and discussion section, data regarding the performance of the proposed method is analyzed, highlighting the most promising results. Lastly, some final remarks are made in the conclusion and future works section.

## 2. Background of Concepts

This section briefly introduces the main concepts needed throughout the rest of the paper. Some image processing and object recognition concepts are discussed, such as Discrete Cosine Transform (DCT), Local Linear Embedding (LLE), and Discrete Wavelet Transform (DWT). For the image pre-processing, Gaussian filters, contrast stretching, cork stopper outline detection for cropping, and bicubic interpolation on rescaling are mentioned. Moreover, due to the need for image rotation correction, some methods such as Radon Transform (RT) and Hough Transform (HT) are discussed.

### 2.1. Bicubic Interpolation on Rescaling

Knowing that the images are presented with different sizes, and as it is necessary to compare them to each other, they must be rescaled to a standard, common size. In this work, the value chosen for their size was 512 × 512 pixels, which also coincides with the maximum image size among the images available in the database. For this rescaling, pixels have to be added by means of interpolation. In order to create a smooth transition from one pixel to another, the intensity of interpolated pixels is calculated based on a cubic interpolation of the values of its neighbors.

### 2.2. Gaussian Filter

Linear filtering, such as that obtained with Gaussian filters, is commonly implemented in computer vision to reduce noise, especially Gaussian filters. In a linear filter, each pixel value is obtained from a linear combination of the intensity values of its neighboring pixels, as seen in Equation (1).

(1) $$L\left(i,j\right)=\frac{1}{N}\;\underset{k,l}{\sum }I\left(i+k,j+l\right)\cdot H\left(k,l\right),$$

where $L\left(i,j\right)$ are the intensities of each individual pixel. For Gaussian filters, the coefficients of the Gaussian filter $H\left(k,l\right)$ are obtained from Equation (2).

(2) $$H\left(k,l\right)=\frac{1}{2\pi \sigma }e^{-\frac{1}{2\sigma^{2}}\left(k^{2}+l^{2}\right)}$$

### 2.3. Cropping

**Figure 1:**
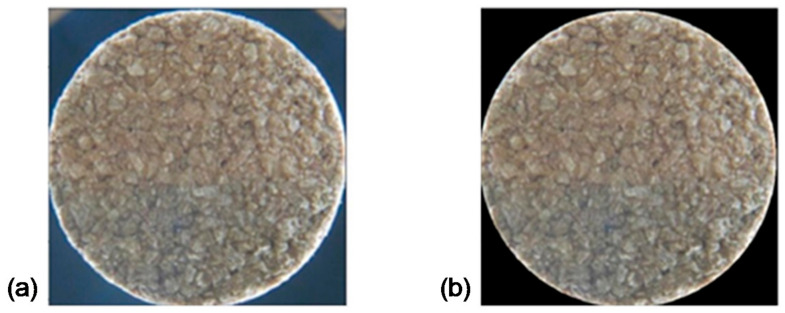
Pre-processed image: (**a**) before the cropping operation; (**b**) after the cropping operation.

For a better evaluation of the hamming distance between the images, their background, which may differ from image to image, needs to be removed. For this, the color histogram is calculated, and the correct brightness value for the identification of the pixels that correspond to the edges is determined. Knowing that there is no guarantee that the pixels identified as the edge of the cork stopper representation are immediately adjacent, an interpolation between a sample of boundary pixels, which are known to be correctly identified, was performed. The radius value is also limited so that the segmentation is correct (Figure 1). By applying the least square method, the center coordinates $C\left(x_{C},y_{C}\right)$ and the radius *R*, values are obtained, thus assigning a null value to the pixels outside the condition shown in Equation (3).

(3) $${\left(x-x_{c}\right)}^{2}+{\left(y-y_{c}\right)}^{2}<R^{2}$$

### 2.4. Contrast Stretching

**Figure 2:**
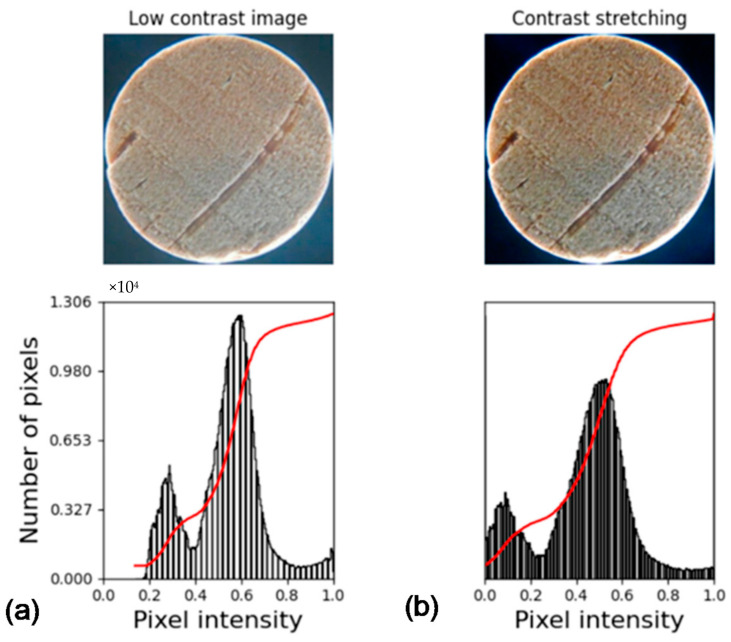
Example of contrast stretching. Image and histogram: (**a**) without stretching; (**b**) with stretching.

The images can be presented with varying brightness depending on the device used in their collection and considering the necessity of comparing them among each other. Therefore, a normalization may be performed. This is done by mapping the values in a low contrast image, for example, to the full length of the [0 to 255] interval, which can be seen in Figure 2.

### 2.5. Discrete Cosine Transform (DCT)

DCT is a method for compression and decompression of an image based on the value of its pixels [16,17,18], transforming it into a sequence of relevant data points. The relevance of these data points is evaluated in terms of the sum of cosine functions with different frequencies. As DCT is more accurate in small images, it is common practice to divide the processed image in N blocks of a b × b size. After this division, the DCT maps the pixel values in those blocks from the spatial domain to the frequency domain [19]. Mathematically, the transformation results from Equation (4),

(4) $$C_{i}\left(u,v\right)\;=\;\frac{a\left(u,v\right)}{N}\cdot \overset{b-1}{\underset{j=0}{\sum }}\overset{b-1}{\underset{k=0}{\sum }}B_{i}\left(j,k\right)\cdot \cos\frac{\left(2j+1\right)u\pi }{2b}\cdot \cos\frac{\left(2k+1\right)v\pi }{2b}$$

where $B_{i}\left(j,k\right)$ is the intensity value of the pixel with coordinates (*j*,*k*) in the block with index I = 1,…, N and $a\left(u,v\right)$ is defined in Equation (5).

(5) $$a\left(u,v\right)\;=\;\left\{\begin{array}{ll} 2, & u,v\neq 0\\ 1, & u,v=0 \end{array}\right.$$

Then, the first M values, where M can vary from 1 to N, are retrieved from a zig-zag sequence. This ensures data compression, as each b × b matrix has been converted into a vector of dimension M. These N vectors are then combined into a single M × N matrix.

### 2.6. Local Linear Embedding (LLE)

To obtain a hash with N-bit as a final result, it is necessary to perform data compression using an efficient method such as local linear embedding [20], an alternative to principal component analysis and multidimensional scaling, which were reported by Tang et al. [21]. This method is performed by first looking for K clusters of points computed from a matrix M × N. These points are normalized and organized according to the minimum distances between them and this is achieved by using a weight matrix. Following this, the eigenvectors are obtained from the weight matrix and a new matrix d × N is attained. In comparison with the initial M × N matrix, the new matrix is more compact.

### 2.7. Discrete Wavelet Transform (DWT)

A wavelet transform is a transformation that projects a given signal into the frequency- and time-domain whose basis functions are scaled and shifted versions of a wavelet. The discrete wavelet transform (DWT) is no more than the implementation of such wavelet transform resorting to a discrete set of the wavelet scales and translations. Therefore, the similarity between the DCT and the DWT is easily recognized. The main difference lies in the fact that the DCT relies on cosine functions of unlimited duration instead of short-duration transient functions (wavelets) for the set of basic functions [22]. Consequently, the DWT performs better extraction of information from high-frequency components which in the case of images corresponds to edges. This was thought to be of importance given the non-smooth characteristics of cork images. For the sake of differentiating the two transforms it may also be added that the DWT provides high resolution in both the time- and frequency-domain, while the DCT only presents high resolution in the frequency-domain [23]. The DWT is also widely used in image processing for compression [24,25] and noise removal [22].

In general terms, the wavelet transform is expressed by Equation (6),

(6) $$\Psi_{s}^{\Psi }\left(\tau ,s\right)\;=\;\frac{1}{\sqrt{\left|s\right|}}\int x\left(t\right)\Psi^{*}\left(\frac{t-\tau }{s}\right)dt$$

where * refers to the complex conjugate and the function *Ψ* is the transforming function (mother wavelet). The transformed signal is a function of *τ* and *s*, corresponding, respectively, to the translation and scale factors.

### 2.8. Difference Hash (DH)

Difference hash is an alternative method to DCT and DWT. In DH, the input image is converted to a 2D grayscale image, downsized to N × N. A row hash is produced with either 1 or 0 values depending on whether pixel intensity is increasing or decreasing from pixel to pixel in the direction of increasing column index. A similar process is done for each column, resulting in a final hash size of 2 × N^2^.

### 2.9. Average Hash (AH)

Similar to DH, the average hash method takes the input image and converts it to grayscale and the image is downsized to N × N. The average intensity of all pixels on the image is then calculated and each pixel is compared to that average value. Following this, depending on whether each pixel has above- or below-average intensity, the final hash is produced.

### 2.10. Radon Transform (RT)

The Radon transform is a technique that allows features within the image to be detected [26] by computing the total density of a function f along a given line S (ρ, θ) [27]. In Equation (7), the Radon transform is defined.

(7) $$Rf\left(\rho ,\theta \right)=\;\int_{-\infty }^{+\infty }f\left(x\left(s\right),y\left(s\right)\right)ds=\;\int_{-\infty }^{+\infty }f\left(\rho \cdot \cos\theta -s\cdot \sin\theta ,\;\rho \cdot \sin\theta +s\cdot \cos\theta \right)ds$$

**Figure 3:**
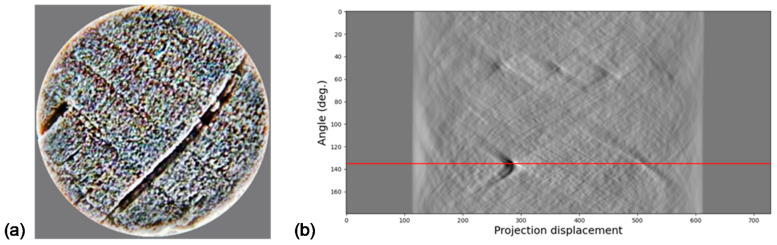
(**a**) Image; (**b**) Radon Transform (RT) application.

This transform has been found to be useful in object and pattern recognition applications [28,29] because, apart from being a lossless transform (meaning information can be accurately reconstructed), it is a transform used for mapping a set of coordinates in the spatial domain to a Radon (parametric) domain. Even more relevant regarding the present work are its properties concerning rotation and scaling transformations [30]. Regarding this, Figure 3 shows how finding the relevant points in a sinogram determines the direction of the image.

### 2.11. Hough Transform (HT)

HT and RT are very similar [31]. For the present work, they both could have been used with the purpose of finding image orientation. However, while Radon transform finds the global image orientation looking for different perspectives, Hough transform is built to find straight lines based on certain features, even in the presence of noise [31]. The versatility of HT was shown by works such as [32], which has applied it in arbitrary curve analysis.

**Figure 4:**
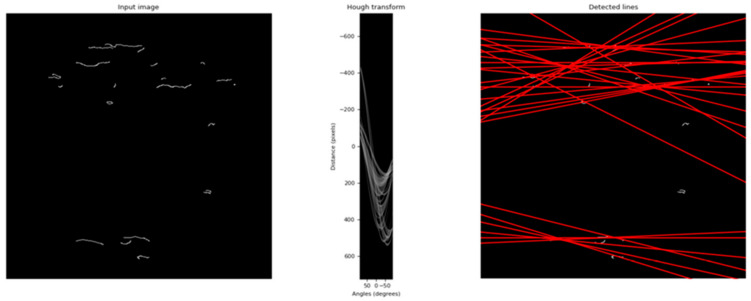
Hough transform application after Canny edge detection.

To detect these features, the conversion from the spatial domain to the parametric domain is done, and a sinogram is obtained. This sinogram (Figure 4) exhibits brighter regions, each of which identifies a dominant direction in the image. These regions of interest (local maxima) are then evaluated by the average and variance values, which are used to characterize, respectively, an approximate value for the rotated angle and the reliability of that approximation.

In the presence of angular data ($a_{1},\ldots ,a_{n})$, the mean M and variation V are determined by Equations (8)–(12).

(8) $$C_{p}=\overset{n}{\underset{i=1}{\sum }}\cos\left(a_{i}\right)$$

(9) $$S_{p}=\overset{n}{\underset{i=1}{\sum }}\sin\left(a_{i}\right)$$

(10) $$\rho =\sqrt{C_{p}^{2}+S_{p}^{2}},T_{p}=\tan^{-1}\left(\frac{S_{p}}{C_{p}}\right)$$

(11) $$M=\left\{\begin{array}{lll} T_{p}, & C_{p}>0\;\wedge & S_{p}>0\\ T_{p}+\pi , & & C_{p}<0\\ T_{p}+2\pi , & C_{p}>0\;\wedge & S_{p}<0 \end{array}\right.$$

(12) $$V=1-\frac{\rho }{n}$$

### 2.12. Hamming Distance

The hamming distance can be interpreted as a norm-1 distance, as seen in Equation (13).

(13) $$∥V-W{∥}_{1}=\overset{n}{\underset{i=1}{\sum }}\left|v_{i}-w_{i}\right|$$

*V* and *W* are now denoted as binary vectors. This distance is commonly interpreted as being the sum of the serial results of the exclusiveness between $v_{i}$ and $w_{i}$, which means that the sum of $v_{i}⊗w_{i}$, *i* = 1,…, n.

In this work, Hamming distance is used to compare two different hashes at a time.

## 3. State of the Art

In this section, the latest developments in image hashing are reviewed.

For image hashing codes generation, various state-of-the-art image hashing methods have been proposed over the last decade. Du et al. [33] defined five types of image hashing methods: invariant feature transform-based methods, local feature points-based method, dimension reduction-based method, statistics features-based methods, and learning-based methods. For invariant feature-related methods, the robustness and major feature extraction are main advantages of this kind of methods. For instance, in order to do hash size reduction based on big blocks in block-based analysis, Yan et al. [34] performed a binary approach to accomplish both the smallest block size and more compact hash, using a Quaternion lowpass filter. Qin et al. [35] used a combination of color feature and local texture extraction, making this a hybrid approach, using Weber local binary pattern. Local feature points methods have the advantage of defining compression in the same way mainly for rotated and non-rotated images, using invariant scalar approaches, which ensure that features are preserved even under image processing. Qin et al. [36] developed a method to attribute a final hash using color vector angle, and it was shown that it is secure even after quantizing and scrambling. Pun et al. [37] designed a method for tampering detection using progressive feature point selection, which filters the key points as opposed to the Scale-Invariant Feature Transform (SIFT) method, and better detects the tamper feature. Dimension reduction-based methods are also compression methods, the difference being that they reduce the dimensional space into a lower one, based on the significance of each dimension. Examples for these are LLE, Singular Value Decomposition (SVD), and Fast Johnson-Lindenstrauss Transform (FJLT). These methods have been successfully applied and improved. For instance, Sun et al. [38] proposed a method based on Fourier-Mellin transform to improve performance under pre-processing (which can be included in local feature points-based methods category) and dimension reduction, using statistic structure and sparse coefficients of wavelet to assure efficiency. Liu et al. [39] tried to do image recovery performance for image authentication and tampering identification. For that, the authors used low-rank representation for obtaining both feature and error matrices and compressive sensing for the obtention of primary features. By having lower error values, this method showed better results than similar hashing methods. Statistics feature-based methods rely on statistic parameters such as mean, variance, or histogram to perform extraction of image features, which are used not only for tackling noise, blur, and distortions but especially for verification of uniqueness of image hashes. Srivastava et al. [40] proposed a robust method for conversion of images into hash codes in the presence of multiple types of attacks, mainly rotation, using color space conversion, Radon transform, and DCT, followed by evaluation thought four statistic parameters mean, standard deviation, skewness, and kurtosis. Huang et al. [41] proposed a method using two different types of features, using DCT coefficients for local features and texture for global features. Here, statistical characteristics were extracted to improve perceptual robustness. At last, learning-based methods use training data in order to tune the algorithm, usually neural networks, for better performing of image authentication tasks. Jiang et al. [42] used deep Convolutional Neural Networks (CNNs) in order to do content authentication. Through data training, image feature matrix was evaluated as the output of neural networks. The results of this paper show that discrimination behavior is better than other methods and robustness is shown to be acceptable. According to Du et al. [33], and despite the need for training data and time computational costs, the learning methods are better in terms of accuracy. Moreover, it can be verified that DCT and Radon transform are two of the most used approaches for image authentication, since a wide range of works use this method and have obtained good results.

## 4. Methodology

The current methodology is based on the implementation of methods taken from the referenced literature in the background of concepts section. Following the implementation, the obtained results were evaluated and, when necessary, improvements were made considering the specifications of the application.

**Figure 5:**
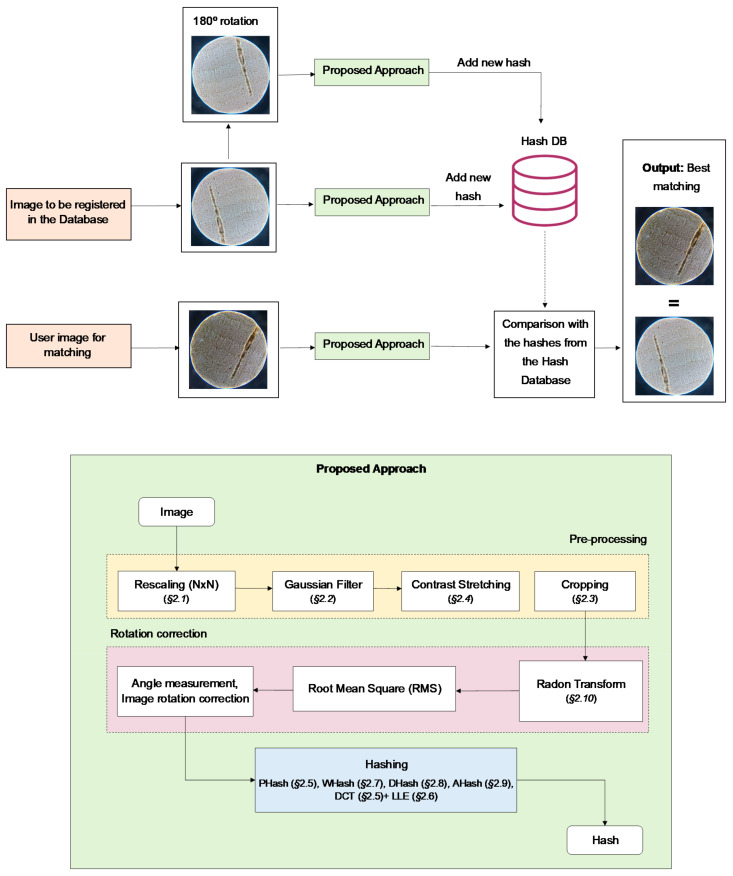
Block diagram of the cork authentication process; proposed hash-based approach.

The entire process, from image acquisition to image matching, can be observed in the theoretical framework, seen in Figure 5. The input image is put through pre-processing, Radon transform, hashing conversion, and is finally compared with the DB (hashes from original and cloned images). A more detailed view of the image-to-hash conversion steps is shown in Figure 5. After retrieving the image, its pre-processing is accomplished by the following sequence of steps: rescaling (Section 2.1), Gaussian filter (Section 2.2), contrast stretching (Section 2.4), and cropping (Section 2.3). Then, as a first step towards rotation correction, Radon transform is applied and a matrix regarding the outputted sinogram is obtained. For each discretized angle, an average of the projection of the pre-processed image along the radius axis is calculated, by means of the Root Mean Square (RMS); the discretized angle for which RMS value is the maximum value is taken to be the correct rotation angle. The rotation is corrected in the pre-processing of the image and then, finally, a hash method converts the image to a codified form.

Since generated hash codes are strongly influenced by rotation, the performance of different rotation measurement and correction algorithms was previously evaluated. Due to the promising results, Radon transform was chosen over Hough transform. Further details on rotation influence and rotation algorithms are shown in the Results section.

However, RT, by itself, was insufficient. RT can rotate an image to the main direction, with no distinction between the two possible solutions (0° and 180°); this concern was minimized with the addition of rotated image duplicates to the database.

The following hash functions were then tested: average hash, wavelet hash, difference hash, and perceptual hash. The wavelet hash code was obtained from the application of DWT, while the perceptual hash code was produced by the application of DCT. Moreover, the method used by Tang et al. [21] (DCT + LLE) was tested using the following adaptations: the steps concerning hash code security were discarded (given the unclonability of cork features); further, the final step in LLE (Section 2.6), low-dimensional embedding vector calculation, was performed numerically with the initialization matrix 1.

The hash codes were obtained with a 256-bits size, with further investigation on hash code size influence. For this purpose, 64- and 1024-bits hash codes were also created. In the results section, time costs for this process will be detailed for the different hashing methods.

For evaluation purposes, a database (DB) [10] was used containing images taken with a phone camera (Sony Z3X1, Tokyo, Japan) and two test sets (TS1 and TS2) containing images taken with two other different cameras (Asus Zenfone 5,Taipei, Taiwan, and Sony PlayStation Eye, respectively). In these tests, each DB hash was compared to every hash in both TS1 and TS2, with the similarity between each hash pair being evaluated by the correspondent hamming distance (HD). The number of pairs for which the HD was lower than the correct match was tallied. This information is taken as a measure of the performance of the matching method. Obviously, a perfect result would translate to no pairs having a smaller HD than the correct pair, i.e., the correct match being associated with the minimum HD.

**Figure 6:**
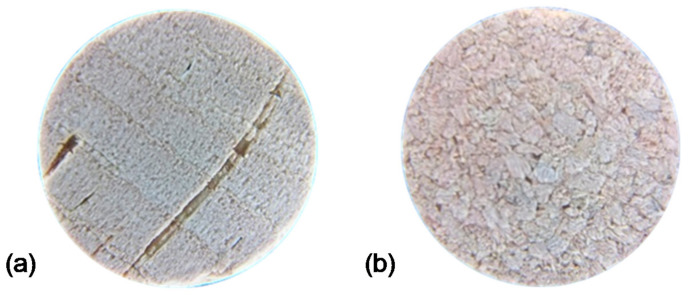
Cork types: (**a**) natural; (**b**) agglomerated.

It is also important to mention that there are two types of corks: agglomerated and natural [43]. Database A is a set of 300 images of the agglomerated type of corks, and databases E and N are sets with 100 images each of the natural type. This difference is portrayed in Figure 6. The distinction between such types of corks is of the utmost importance when designing the image processing method, especially when it comes to rotation correction. In the results section, only the natural type is discussed, due to the complexity of the image correction problem for agglomerated corks.

## 5. Results and Discussion

The results for the applied correspondence methods are presented in this section: firstly, a comparison of images is done without any pre-processing or previous rotation correction; thus, these corrections are considered; in the last subsection, time performance of these methodologies is addressed, with further discussion.

As mentioned in the methodologies, there are several steps that are sequentially used. It is important to evaluate how influential each one of those steps is on the final success rates. In an initial approach, a study was performed in order to assess the importance of the application of Radon transform to correct the rotation of cork images. The parameter values adopted are listed and associated with the corresponding methods (identified in Section 2) in Table 1.

**Figure A1:**
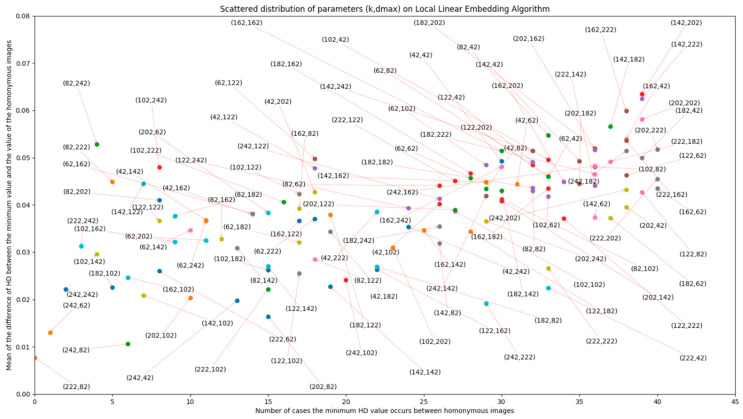
Number of correct matches (per 60 possible pairs) depending on the LLE parameter variation and the difference between minimum value and value for homonymous images.

**Figure A2:**
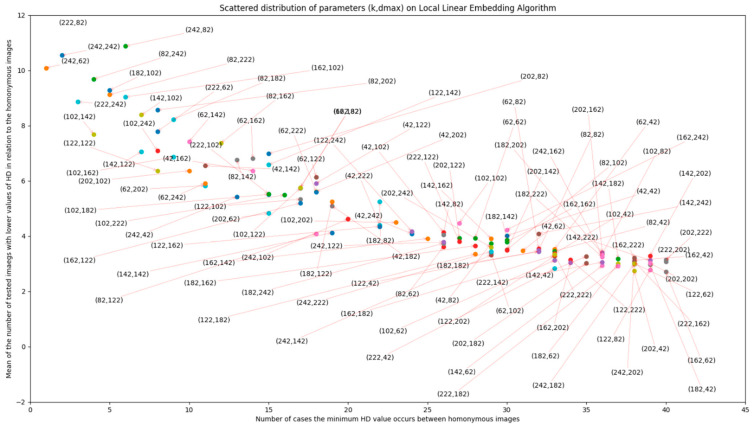
Number of correct matches (per 60 possible pairs) depending on the LLE parameter variation and the number of tested images with lower values of HD in relation to homonymous images.

DCT + LLE, LLE parameters have been proven to have a relevant role in matching accuracy, as evidenced by Figure A1 and Figure A2 in Appendix A. In these figures, scatter plots are presented, in which LLE parameters k and dmax vary according to the different perspectives of evaluation. It is important to minimize hamming distance (HD) of the correct match and increase the value of HD for non-homonymous images, in order to decrease the number of false positives returned by the method. With these objectives in mind, the values for these parameters were made to vary between 42 and 242, with an increment of 20, for 20 images extracted from the database (giving 60 possible homonymous pairs). The lowest limit of the variation interval (42) was chosen because it was found to be close to the lowest possible value for these parameters that still allows for information about the image to be preserved. From the plots, it is concluded that the pair (k, dmax) that fits the best performance would be (202,222). Further evaluation of these effects (with smaller increments in parameter variation) should be addressed in future works.

To analyze the efficiency of the methods composed by the hash function, Radon transform, and 180° rotation duplicates, three hash test sets were considered. The different test sets are useful for identifying the influence of each stage of the method on its success rate. These hash test sets are as follows:

S1: Hash codes from all images in the database
S2: Hash codes from images successfully rotated by RT (0° or 180°)
S3: Hash codes from images successfully rotated by RT to the correct side (0°)

Note that S2 and S3 are smaller than S1. More precisely, their sizes are 85% and 25.5% of S1, respectively.

The percentage of images for which the correct match falls within top 1, top 5, top and Top 10 (also called success rate) was obtained for each hash test set and for each hash function. The top 5 and top 10 results are of interest, given the expectation of higher success rates than those achieved by the top 1 research while also considerably reducing the search space, allowing for algorithms, such as the one presented by [10] to be applied in a timely manner. They display the values of the minimum success rates between both TS1 and TS2 when matched with the DB, for a specific case and method. These top 1, top 5, and top 10 results are presented in Table A1, Table A2, Table A3, Table A4, Table A5, Table A6, Table A7, Table A8, Table A9 and Table A10 (in Appendix B). Due to the need for comparison between image sets that have different sizes, in which case relative values are more suitable, relative measurements were preferred for the accumulative graphic plots presented throughout this section. These plots were obtained in order to show the accumulative number of correct matches achieved as the Top X criteria grows larger and less strict (i.e., X increases).

This section is structured as follows: Section 5.1 shows the results obtained using the adopted and adapted hashing method without any rotation correction; Section 5.2 shows the results for different tries on rotation correction; Section 5.3 refers to the results concerning image rotation correction and contrast stretching and its effect on the previously implemented hashing algorithm for the different sets of images S1, S2, and S3; in Section 5.4, time performance values are displayed for different methods and different sets.

### 5.1. Testing Methods without Pre-Processing 

In a first approach, the performed tests were mainly concerned with the distribution of HD between image pairs. These pairs can be made up of homonymous images or non-homonymous images. Homonymous images are defined as images representing the same cork stopper but photographed with different cell phone cameras. The name “homonymous” was chosen because photograph files of the same cork stopper appear with the same label in the database. The value of HD was also evaluated for various non-homonymous pairs. The algorithm assumes the correct match to be that with the minimum HD; therefore, it was essential to know how many wrong matches had a lower HD than the correct one. Ideally, the expected value for this distribution would be very close to 0, which would mean, in most cases, the homonymous image would also be the image with the minimum HD value. Since this is not the case, considering the high number of images with the value of HD below the value of the homonymous image, the cause of this observation was sought.

**Figure 7:**
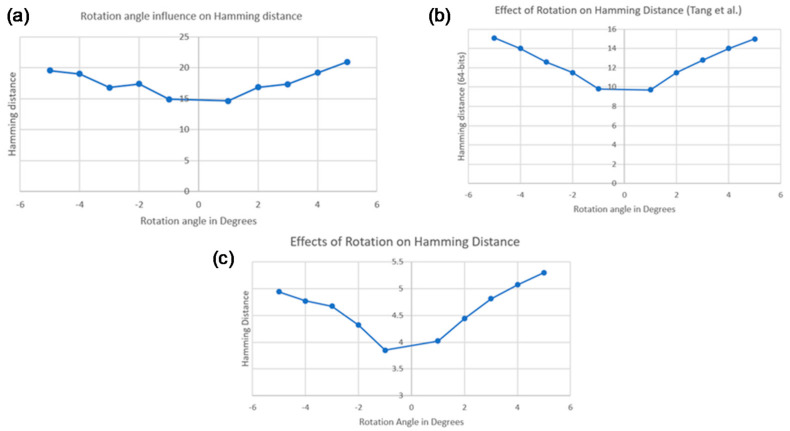
Effect of rotation on hamming distance: (**a**) for a ‘non-cork’ database; (**b**) for a similar ‘non-cork’ database [21]; (**c**) for a cork database.

It is mentioned by [21] that the hamming distance values might be influenced by image rotation. Thus, this hypothesis was tested. An initial test was carried out on a set of 47 images from the same image pack used by the authors [37], and the variation of HD with the angle of rotation was plotted. As can be seen in Figure 7, the results are similar to those presented in that work.

At a later stage, the method was applied to the database to extend the conclusions already drawn, now showing the sensitivity to the rotation for the images of the stoppers. It is possible to verify, in Figure 7, that, although the values of hamming distance tend to decrease with the decrease of the rotation angle, the “linearity” is not so visible. It must be noted that both the values and the variation of these values are much lower than those seen in that work, therefore, the referred discrepancy can be justified by this behavior. The differences between the two plots can be explained by the fact that all the images in the cork database are very similar to each other, which was not the case for the database used in the aforementioned study.

At this point, the limitations of implementing this method for the cork database were identified and it was confirmed that hash codes are sensitive to rotation, especially in images such as the ones being studied (cork stoppers).

It was also noticed that some images have characteristics that allow for rotation angle determination, such as type E and N corks, which show natural lines in the same direction. After correcting rotation, the adopted method can be successfully applied.

#### General Results and Comparison for the Different Methods

**Figure 8:**
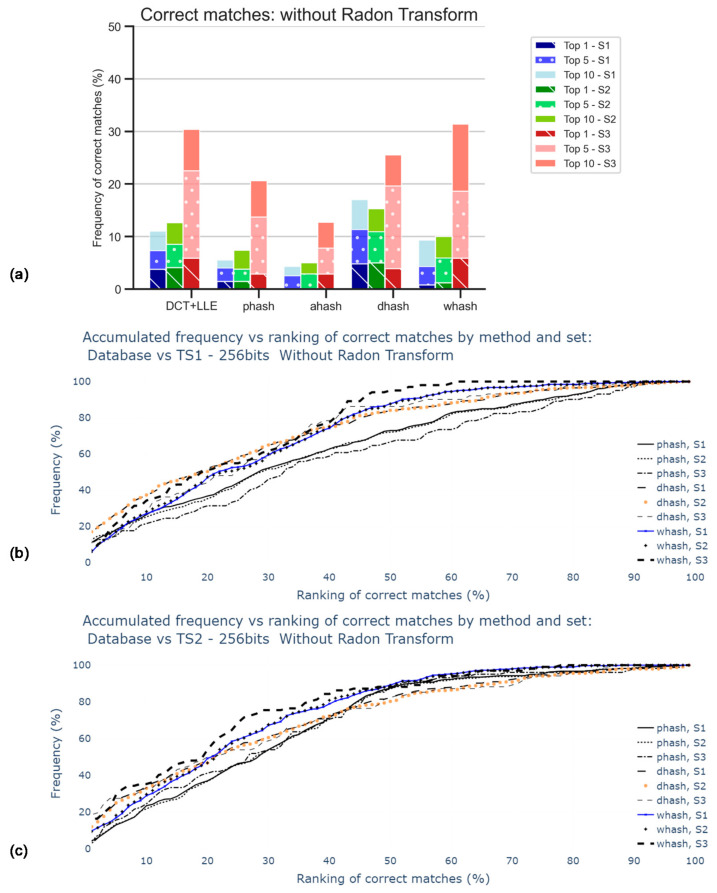
Percentage of correct matches without the application of Radon transform nor contrast stretching: (**a**) for the worst cases between TS1 and TS2 comparisons and only for top 1, top 5, and top 10; (**b**) for the comparison with TS1, all rankings (in percentage); (**c**) for the comparison with TS2, all rankings (in percentage).

The results displayed in Figure 8 are obtained when retrieving 64-bits hash codes using the DCT + LLE method but for adequate comparison with the results presented in Section 5.3, 256-bits hash codes were generated.

Figure 8 is subdivided into three different bars for each hash method, related to the different sets, already mentioned (S1, S2, S3). In this stage, the RT was not applied. The main goal was to obtain the influence of the size of the sets. As observed in the accumulative graphic plots, this influence was not significant since all curves increased in a similar manner. Note that the percentage of accuracy was expected to be apparently better for S3, which was the smallest set among those considered, because the number of images considered for any Top X was independent of the size of the respective set. For Top 1, DH was the method that better performs, but only about 5% in 300 images are correctly matched. AH was the worst method in the tests.

These poor results, displayed in Figure 8a, were indicative of the need for corrective action in the pre-processing stage, i.e., rotation correction and contrast stretching.

### 5.2. Rotation Correction

#### 5.2.1. Rotation Correction Using Hough Transform Operation

Given the already referred similarity between Hough transform and Radon transform, and the fact that they are commonly associated to each other in a wide range of works, some test were run to evaluate the performance of HT for the rotation correction of the stopper cork images.

**Figure 9:**
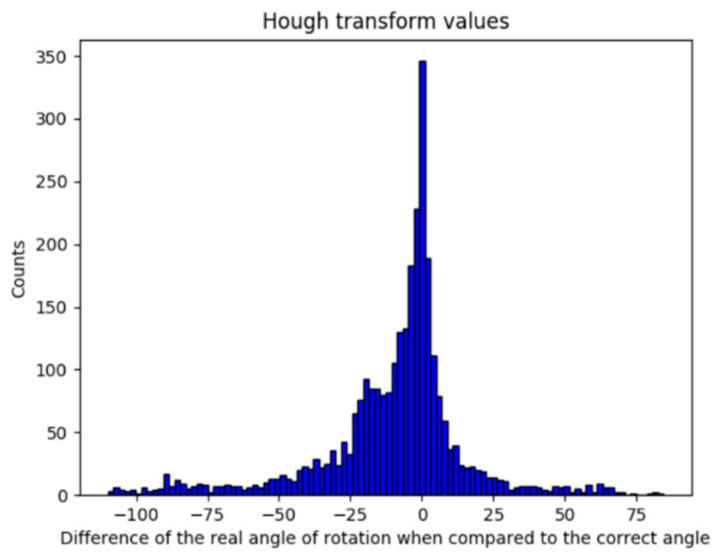
Error obtained by the implementation of Hough transform.

By implementing HT, the results shown in Figure 9 are consistent with the expected behavior, although these were not promising. It was shown in Section 5.1 that a deviation of less than 1° is acceptable; thus, it can be observed that the deviation value (μ = −9.8022; σ = 25.6619) obtained with HT implementation is not sufficiently accurate.

#### 5.2.2. Rotation Correction Using Radon Transform

**Figure 10:**
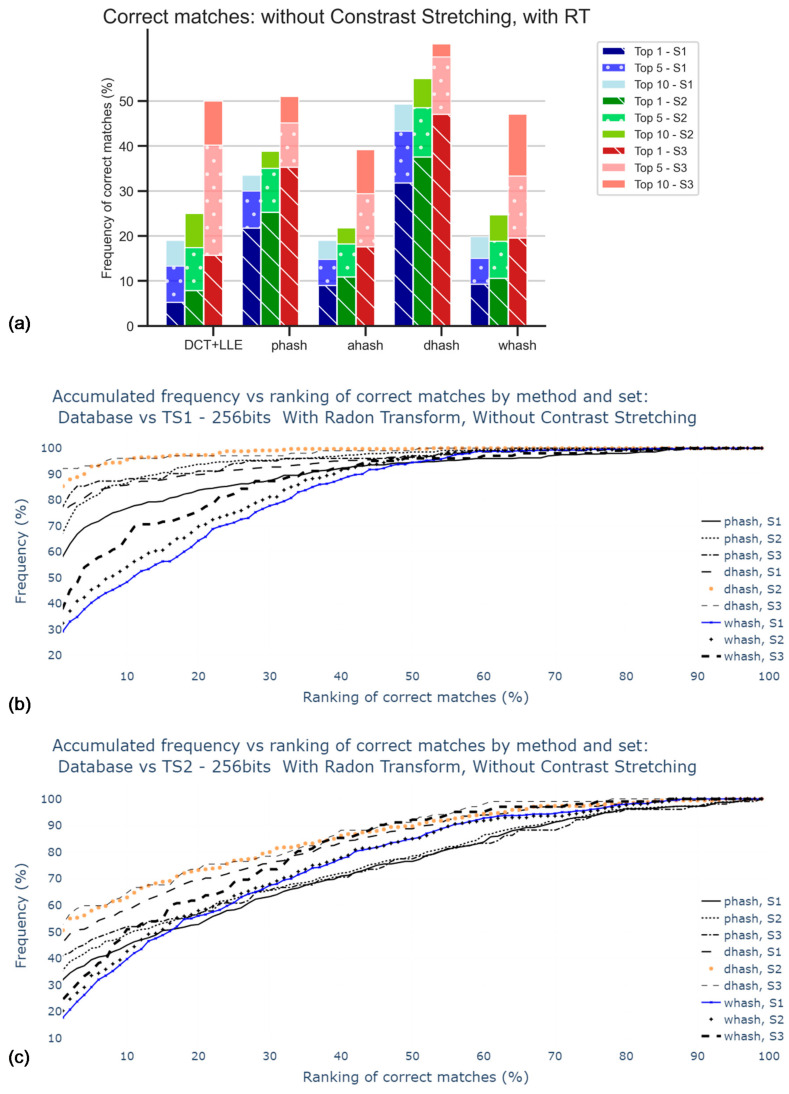
Percentage of correct matches without application of contrast stretching: (**a**) for the worst cases between TS1 and TS2 comparisons and only for top 1, top 5, and top 10; (**b**) for the comparison with TS1, all rankings (in percentage); (**c**) for the comparison with TS2, all rankings (in percentage).

With the implementation of Radon transform, results, displayed in Figure 10, are shown to be better when compared to those in the previous analysis. The dHash was found to be the method that performs more accurately, with almost 30% of correct matches for Top1 of S1, against almost 5% in the case where RT was not applied. Notwithstanding, 30% is still not significantly accurate, which calls for the search of a better approach, such as the equalization of image colors.

### 5.3. Contrast Stretching and Hash sizes

**Figure 11:**
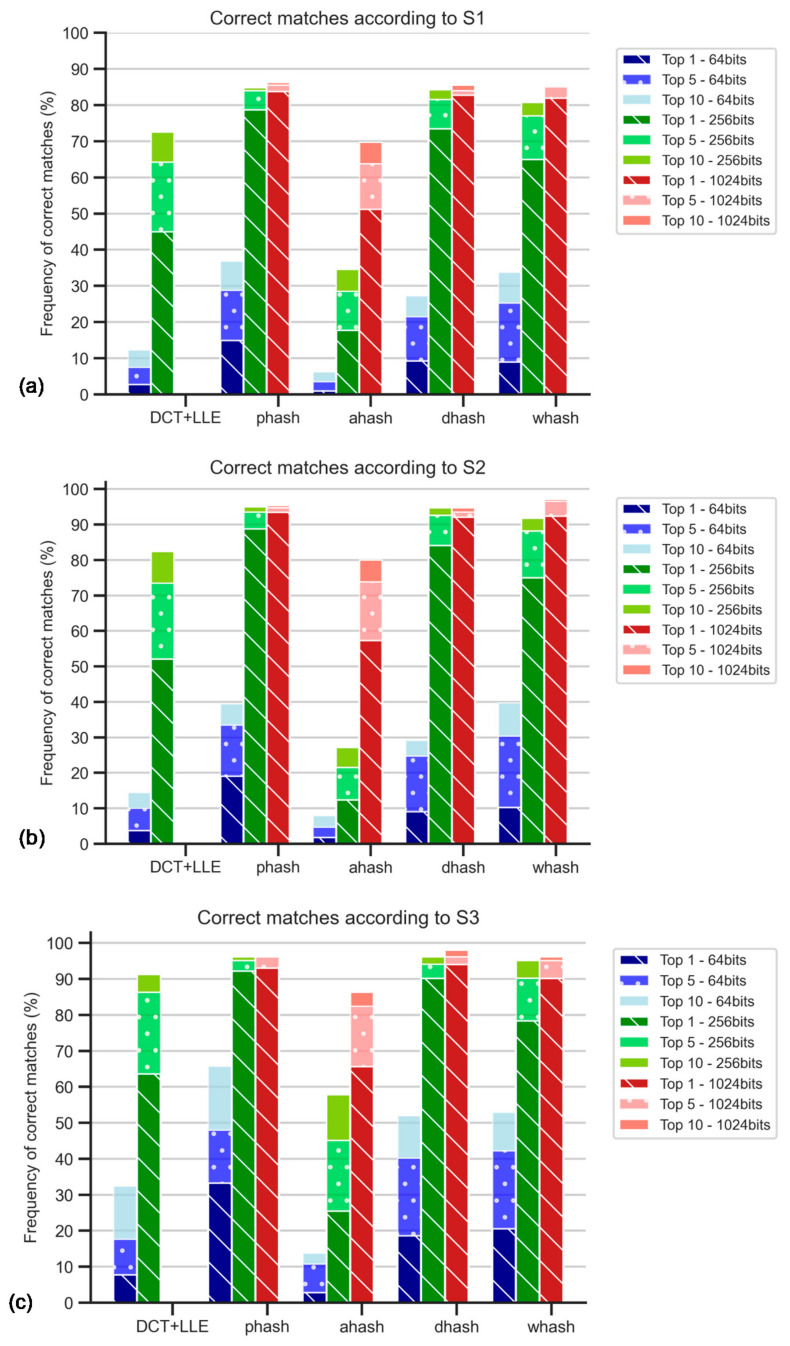

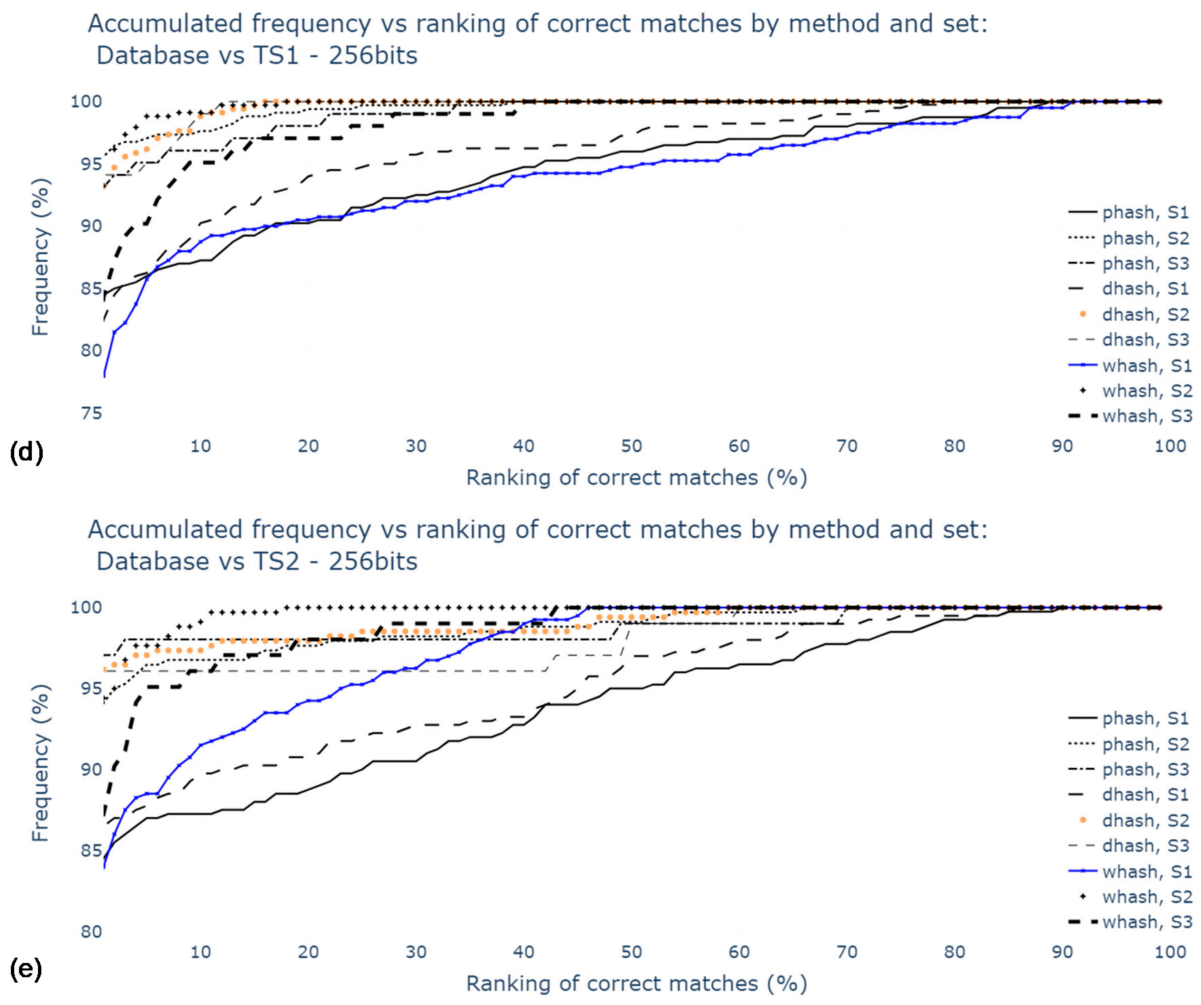
Percentage of correct matches with Radon transform and contrast stretching: for the worst cases between TS1 and TS2 comparisons and only for top 1, top 5, top 10: (**a**) using S1; (**b**) using S2; (**c**) using S3; (**d**) for the comparison with TS1, all rankings (in percentage); (**e**) for the comparison with TS2, all rankings (in percentage).

Figure 11 shows the results for datasets with hashes from original and duplicated images. The values are higher for S2 and S3 results, suggesting that Radon transform and 180° rotation duplicates have a significant influence on matching success. Moreover, pHash and DH were the best methods in the scope of this study. The top 5 and top 10 of S3 with DH method resulted in the best success rate, with 98.0% of correct matches.

WHash and DCT + LLE methods are generally worse than the latter methods; still, wHash can be used as an alternative to pHash and DH, since in some cases, for example, in top 10 of S1, the matching success rates are similar. AH has been promptly identified as the worst method among those studied.

Regarding hash size, it is possible to conclude that the 1024-bit hashes provide higher accuracy, although the use of 256-bit hash codes leads to similar results. Increasing the hash size even further to 4096-bit would be worse, as the value of accuracy would fall to 19.5% for S1, for example. This is due to an increase in sensitivity to noise.

### 5.4. Time Performance

One of the main focuses of the present work was, in addition to the correction for rotation effects, to decrease the time cost of search operations, as suggested by [10]. Thus, an evaluation of the time performance of each of the proposed methods is essential.

In order to observe the differences in time costs between the different steps of the algorithm, it was divided into 5 main steps:

- Step 1: Image acquisition
- Step 2: Pre-processing (Gauss filter, contrast stretching, cubic interpolation)
- Step 3: Radon transform and angle calculation
- Step 4: Image-to-hash conversion
- Step 5: Hamming distance and final correspondence

The values for estimated time performance can be seen in Table 2 and Table 3, for a computer with processor Intel^®^ Celeron^®^ N4000 CPU @ 1.10 GHz, Windows 10 Operative System and 4 GB RAM. In Table 2, distributions of time (average—μ, standard deviation—σ) are displayed for Steps 1–3 and 5 and in Table 3 for Step 4, different methods used and hash different sizes.

It is possible to conclude that Radon transform is the step of the algorithm that takes longer to execute, with almost 8 s required per image. This suggests that the other times are reduced in comparison. It is likely that vectorial computations greatly reduce this time. Sample testing using MATLAB 2020b, where RT is vectorially performed hints at a more than tenfold performance improvement. These preliminary results came from a 64-bit Windows 10 SO with processor Intel^®^ Core™ i7-10510U CPU @ 1.80GHz 2.30 GHz, achieving an RT run time of $\left(\mu ,\sigma \right)\;=\;\;\left(0.1579,\;0.0103\right)\left(s\right)$ on MATLAB 2020b against $\left(\mu ,\sigma \right)\;=\;\left(1.7786,\;0.0247\right)$(s) for Python 3.7 with this same hardware were approximately 11.3 times smaller. Concerning Step 4, DCT + LLE seems to be the worst method when it comes to time performance, for the same reason that was pointed out for the RT step, i.e., the non-vectorial form. DH seems to be the fastest method; however, apart from the DCT + LLE, all methods were relatively fast. When compared to the literature [44], this work seems to be a faster way of obtaining correspondence, requiring 0.241 ms for comparison of 100 HD when compared to more than 9 ms occurred in work [44]. The times were measured on the same computer and, as such, the mentioned values are comparable. The values in Table 4 show that the present approach is almost 40× faster than that work.

## 6. Conclusions and Future Work

Hash codes have been successfully used for efficient natural cork stoppers image matching, being of paramount importance in cork stopper authentication for anticounterfeiting purposes.

The proposed approach is centered on Radon transform to correct image rotation and allow the generation of meaningful image hashes that later can be used for image comparison. The best result was obtained using a hash size of 1024-bit and DH, achieving a success rate of 98% for a top 10 approach and taking only 2.41 μs per match, which was much better than [44]. This result demonstrates that the proposed approach is valuable for the mentioned case study of natural cork image comparison; this approach is likely to be useful for any image with a dominant direction.

Since the agglomerated corks do not possess the directionality needed for the RT to operate, the development of rotation correction methods is still necessary. Future research might involve the approach proposed in [45,46] regarding isotropic or rotation invariant textures.

## Figures and Tables

**Table 1:** List of parameter values.

Section 2	Parameter Designation	Value
Section 2.1	Image size	512
Section 2.2	Lowpass filtering window size	3
Section 2.2	Standard deviation	8
Section 2.3	Rad. Minimum limit value	230
Section 2.3	Rad. Maximum limit value	260
Section 2.3	Center min. Limit value	230
Section 2.3	Center max. Limit value	280
Section 2.4	Clip value	0.03
Section 2.5	B (N = 64-bit hash code)	64
Section 2.5	B (N = 256-bit hash code)	32
Section 2.5	B (N = 1024-bit hash code)	16
Section 2.6	K (N = 64-bit hash code)	15
Section 2.6	Dmax (N = 64-bit hash code)	40
Section 2.6	K (N = 256-bit hash code)	42
Section 2.6	Dmax (N = 256-bit hash code)	42
Section 2.6	K (N = 1024-bit hash code)	400
Section 2.6	Dmax (N = 1024-bit hash code)	450

**Table 2:** Distribution of computational times for all steps excluding Step 4.

Step 1		Step 2		Step 3		Step 5	
μ (s)	σ (s)	μ (s)	σ (s)	μ (s)	σ (s)	μ (s)	σ (s)
0.0243	0.0131	0.8178	0.0765	7.8382	0.4197	0.0035	0.0065

**Table 3:** Distribution of computational times for Step 4.

	64-bit		256-bit		1024-bit	
	μ (s)	σ (s)	μ (s)	σ (s)	μ (s)	σ (s)
pHash	0.0079	0.0250	0.0076	0.0192	0.0113	0.0252
aHash	0.0071	0.0377	0.0113	0.0732	0.0086	0.0156
wHash	0.0774	0.0235	0.1010	0.0420	0.1330	0.6917
dHash	0.0041	0.0057	0.0050	0.0108	0.0065	0.0055
DCT + LLE	17.3090	1.9727	19.4306	0.5628	274.3112	83.3862

**Table 4:** Comparing times for different works (equivalent to Step 5).

Method	Literature [44], CBIR (ms)	Current Work, Hashing (ms)	Ratio
Average of Comparing Time	9.6	0.241	39.83

## Appendix B. Tables of Accuracy Results

**Table A1:** Correct matches results for database vs. TS1 comparison—without Radon transform or contrast stretching application (256-bit).

		DCT + LLE	pHash	aHash	dHash	wHash
S1	Top 1	6.8%	6.5%	1.3%	5.0%	0.8%
	Top 5	12.3%	10.8%	3.3%	14.8%	4.3%
	Top 10	16.0%	12.3%	5.0%	21.0%	9.3%
S2	Top 1	8.5%	7.1%	1.2%	6.2%	1.2%
	Top 5	13.8%	11.5%	4.1%	17.4%	5.9%
	Top 10	20.3%	14.7%	5.0%	23.5%	10.0%
S3	Top 1	11.8%	7.8%	2.9%	3.9%	5.9%
	Top 5	23.5%	13.7%	7.8%	19.6%	18.6%
	Top 10	30.4%	20.6%	12.7%	25.5%	31.4%

**Table A2:** Correct matches results for database vs. TS2 comparison—without Radon transform or contrast stretching application (256-bit).

		DCT + LLE	pHash	aHash	dHash	wHash
S1	Top 1	3.8%	1.5%	0.0%	4.8%	3.8%
	Top 5	7.3%	4.0%	2.5%	11.3%	9.0%
	Top 10	11.0%	5.5%	4.3%	17.0%	10.8%
S2	Top 1	4.1%	1.5%	0.0%	5.0%	4.1%
	Top 5	8.5%	3.8%	2.9%	10.9%	8.8%
	Top 10	12.6%	7.4%	5.0%	15.3%	11.5%
S3	Top 1	5.9%	2.9%	2.9%	12.7%	11.8%
	Top 5	22.5%	13.7%	9.8%	24.5%	22.5%
	Top 10	35.3%	22.5%	20.6%	37.3%	35.3%

**Table A3:** Correct matches results for Database vs TS1 comparison—with Radon transform or without contrast stretching application (256-bit).

		DCT + LLE	pHash	aHash	dHash	wHash
S1	Top 1	5.3%	21.8%	9.0%	31.8%	9.3%
	Top 5	13.3%	30.0%	14.8%	43.3%	15.0%
	Top 10	19.0%	33.5%	19.0%	49.3%	19.8%
S2	Top 1	7.9%	25.3%	10.9%	37.6%	10.6%
	Top 5	17.4%	35.0%	18.2%	48.5%	18.8%
	Top 10	25.0%	38.8%	21.8%	55.0%	24.7%
S3	Top 1	15.7%	35.3%	17.6%	47.1%	19.6%
	Top 5	40.2%	45.1%	33.3%	59.8%	33.3%
	Top 10	50.0%	51.0%	42.2%	62.7%	47.1%

**Table A4:** Correct matches results for Database vs TS2 comparison—with Radon transform or without contrast stretching application (256-bit).

		DCT + LLE	pHash	aHash	dHash	wHash
S1	Top 1	15.8%	45.3%	12.8%	62.8%	15.3%
	Top 5	30.3%	54.8%	16.8%	74.3%	25.5%
	Top 10	37.8%	61.8%	20.3%	77.3%	31.5%
S2	Top 1	20.6%	53.8%	15.6%	72.4%	17.9%
	Top 5	37.9%	66.2%	20.9%	84.7%	30.9%
	Top 10	48.2%	72.9%	24.7%	87.9%	37.1%
S3	Top 1	33.3%	72.5%	18.6%	88.2%	32.4%
	Top 5	52.9%	85.3%	29.4%	93.1%	53.9%
	Top 10	67.6%	87.3%	39.2%	96.1%	61.8%

**Table A5:** Correct matches results for database vs. TS1 comparison—64 bits.

		DCT + LLE	pHash	aHash	dHash	wHash
S1	Top 1	3.3%	16.5%	1.3%	9.3%	15.5%
	Top 5	11.5%	28.8%	3.5%	21.5%	27.5%
	Top 10	17.8%	37.5%	6.3%	29.0%	37.3%
S2	Top 1	3.8%	20.0%	1.8%	10.9%	17.9%
	Top 5	14.1%	35.0%	4.7%	25.3%	32.1%
	Top 10	20.3%	45.0%	7.9%	34.4%	43.5%
S3	Top 1	7.8%	33.3%	2.9%	18.6%	20.6%
	Top 5	17.6%	48.0%	10.8%	40.2%	42.2%
	Top 10	35.3%	65.7%	13.7%	52.0%	52.9%

**Table A6:** Correct matches results for database vs. TS2 comparison—64 bits.

		DCT + LLE	pHash	aHash	dHash	wHash
S1	Top 1	2.8%	15.0%	1.0%	9.5%	9.0%
	Top 5	7.5%	28.8%	4.0%	22.0%	25.3%
	Top 10	12.3%	36.8%	7.5%	27.3%	33.8%
S2	Top 1	4.1%	19.1%	1.8%	9.1%	10.3%
	Top 5	10.0%	33.5%	5.3%	24.7%	30.3%
	Top 10	14.4%	39.4%	10.6%	29.1%	39.7%
S3	Top 1	7.8%	36.3%	4.9%	24.5%	23.5%
	Top 5	27.5%	58.8%	13.7%	45.1%	48.0%
	Top 10	32.4%	73.5%	24.5%	66.7%	58.8%

**Table A7:** Correct matches results for database vs. TS1 comparison—256 bits.

		DCT+LLE	pHash	aHash	dHash	wHash
S1	Top 1	48.5%	78.8%	17.8%	73.5%	65.0%
	Top 5	67.0%	84.0%	28.5%	81.5%	77.0%
	Top 10	73.0%	84.8%	34.5%	84.3%	80.8%
S2	Top 1	57.6%	90.3%	12.4%	84.1%	75.0%
	Top 5	76.2%	95.3%	21.5%	92.6%	88.2%
	Top 10	82.4%	96.5%	27.1%	94.7%	91.8%
S3	Top 1	70.6%	92.2%	25.5%	90.2%	78.4%
	Top 5	87.3%	95.1%	45.1%	93.1%	90.2%
	Top 10	91.2%	96.1%	57.8%	98.0%	95.1%

**Table A8:** Correct matches results for database vs. TS2 comparison—256 bits.

		DCT + LLE	pHash	aHash	dHash	wHash
S1	Top 1	45.0%	78.8%	22.5%	76.5%	68.3%
	Top 5	64.3%	84.0%	32.5%	86.5%	79.3%
	Top 10	72.5%	85.0%	37.3%	86.8%	82.3%
S2	Top 1	52.1%	88.8%	16.8%	87.1%	77.6%
	Top 5	73.5%	93.5%	22.4%	96.2%	88.5%
	Top 10	82.9%	95.0%	27.9%	96.5%	92.4%
S3	Top 1	63.7%	96.1%	34.3%	93.1%	84.3%
	Top 5	86.3%	98.0%	50.0%	96.1%	94.1%
	Top 10	94.1%	98.0%	60.8%	96.1%	96.1%

**Table A9:** Correct matches results for database vs. TS1 comparison—1024 bits.

		DCT + LLE	pHash	aHash	dHash	wHash
S1	Top 1	0.5%	85.3%	51.8%	85.0%	82.0%
	Top 5	0.5%	85.8%	63.8%	86.3%	85.0%
	Top 10	7.0%	86.5%	69.8%	87.0%	85.3%
S2	Top 1	0.6%	97.1%	59.4%	96.5%	93.2%
	Top 5	0.6%	97.4%	73.8%	98.2%	96.8%
	Top 10	8.2%	97.9%	80.0%	98.5%	97.1%
S3	Top 1	5.4%	93.1%	65.7%	98.0%	90.2%
	Top 5	14.3%	96.1%	82.4%	98.0%	95.1%
	Top 10	21.4%	97.1%	86.3%	98.0%	96.1%

**Table A10:** Correct matches results for database vs. TS2 comparison—1024 bits.

		DCT + LLE	pHash	aHash	dHash	wHash
S1	Top 1	0.3%	83.8%	51.3%	82.8%	82.3%
	Top 5	0.5%	85.5%	64.5%	84.0%	86.5%
	Top 10	0.5%	86.3%	70.0%	85.5%	87.5%
S2	Top 1	0.3%	93.5%	57.4%	92.1%	92.4%
	Top 5	0.6%	94.7%	73.8%	93.5%	96.5%
	Top 10	0.9%	95.3%	80.9%	94.7%	97.1%
S3	Top 1	0.0%	96.1%	76.5%	94.1%	91.2%
	Top 5	0.0%	96.1%	84.3%	96.1%	96.1%
	Top 10	0.0%	96.1%	88.2%	98.0%	96.1%
